# Simultaneous improvement of alopecia universalis and atopic dermatitis in a patient treated with a JAK inhibitor

**DOI:** 10.1016/j.jdcr.2017.12.016

**Published:** 2018-06-02

**Authors:** Gabriela M. Morris, Zachary P. Nahmias, Brian S. Kim

**Affiliations:** a Division of Dermatology, Department of Medicine, Washington University School of Medicine, St Louis, Missouri; b Center for the Study of Itch, Washington University School of Medicine, St Louis, Missouri; c Department of Anesthesiology, Washington University School of Medicine, St Louis, Missouri; d Department of Pathology and Immunology, Washington University School of Medicine, St Louis, Missouri

**Keywords:** alopecia universalis, atopic dermatitis, JAK inhibitor, tofacitinib, AD, atopic dermatitis, AU, alopecia universalis, IFN, interferon, IL, interleukin, ILCs, innate lymphoid cells, Th2, T-helper type 2 cells

## Introduction

Alopecia universalis (AU) is a severe form of hair loss associated with significant psychological distress. Studies have shown that a predominantly type 1 inflammatory process characterized by the production of interferon (IFN)-γ–producing T cells underlies AU. Atopic dermatitis (AD) is a chronic and relapsing inflammatory skin disease associated with severe pruritus. In contrast to AU, a predominantly type 2 inflammatory response underlies the pathogenesis of AD through the recruitment of T-helper type 2 (Th2) cells, basophils, and group 2 innate lymphoid cells (ILC2s) that collectively produce the effector type 2 cytokines, interleukin (IL)-4 and IL-13.^1^, ^2^ We report a case highlighting the effectiveness of the JAK inhibitor, tofacitinib, in the simultaneous treatment of both AU and AD.

## Case

**Fig 1:**
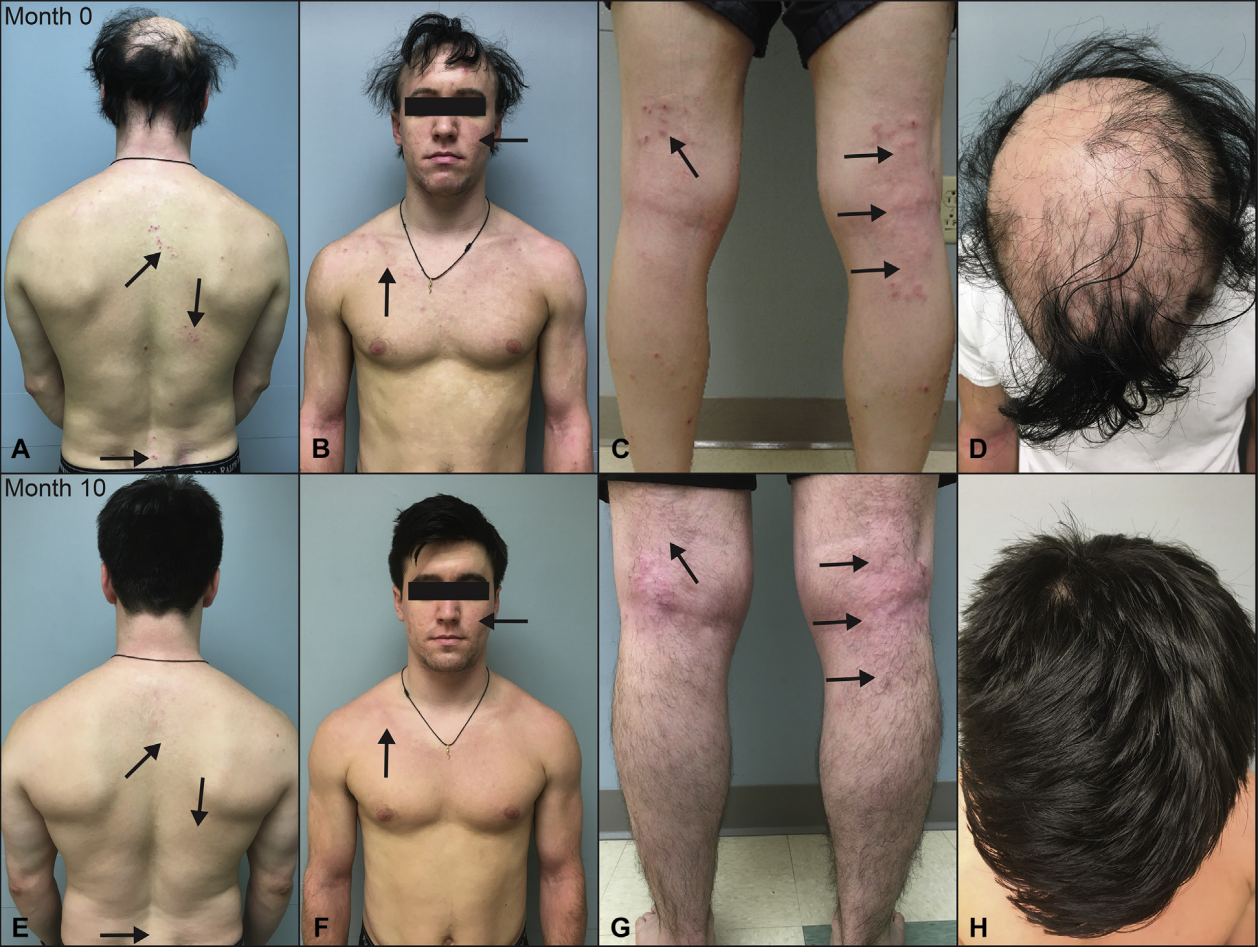
**A**, Posterior view of upper body. **B**, Anterior view of the upper body. **C**, Posterior view of the lower extremities. **D**, Downward view of the scalp before tofacitinib treatment (month 0). **E**, Posterior view of the upper body. **F**, Anterior view of the upper body. **G**, Posterior view of the lower extremities. **H**, Downward view of the scalp after tofacitinib treatment (month 10). Arrows indicate foci of AD lesions.

A 22-year-old man with a history of AU and moderately severe AD (Investigator's Global Assessment score of 3) presented to the clinic for treatment. The patient had a history of AD since childhood with more recent onset AU that progressed in the last 5 years. Physical examination at presentation demonstrated multiple eczematous patches affecting his face, back, chest, and bilateral upper and lower extremities (Fig 1, *A-D*). The patient's itch severity based on the numerical rating scale itch score was 8 (of 10). He also exhibited patches of hair loss on the scalp, eyebrows, eyelashes, face, chest, and bilateral upper and lower extremities (Fig 1, *A-D*). Skin biopsy results of the scalp were consistent with those of AU, which was previously treated with intralesional steroids, methotrexate, and mycophenolate mofetil with minimal improvement. Despite treatment with topical steroids, H1 and H2 antihistamines, and phototherapy for his AD, his condition remained refractory. Additionally, his AD also did not improve while receiving methotrexate and mycophenolate mofetil for his AU. Because of the lack of response of both AU and AD to multiple systemic therapies, the patient was started on off-label tofacitinib at a dose of 5 mg orally, twice daily. After 10 months of treatment, the patient experienced hair regrowth on all of the affected body parts with subsequent improvement of his AD (Fig 1, *E-H*). After treatment, the patient reported a numerical rating scale itch score of 3. Importantly, no adverse effects were reported in terms of clinical symptoms and abnormal laboratory tests.

## Discussion

Tofacitinib is a JAK inhibitor and therefore acts to suppress inflammation by inhibiting multiple cytokine pathways. Here we describe a case of AU associated with moderately severe AD in which tofacitinib simultaneously improved both conditions.

The mechanism by which tofacitinib improves AU has been unveiled through murine models and studies in patients. In this setting, it was found that the production of IL-15 from the outer root sheath of the hair follicle results in the recruitment of CD8^+^ cytotoxic T cells and increased levels of IFN-γ.^3^ Given that both IL-15 and IFN-γ depend on JAK-STAT signaling, disruption of these pathways are likely critical mechanisms by which tofacitinib is effective in AU.^4^

In the context of AD, the type 2 cytokines IL-4 and IL-13 promote skin inflammation as evidenced by the efficacy of dupilumab, a monoclonal antibody targeting the shared IL-4Rα.^5^, ^6^, ^7^ These cytokines depend on JAK-STAT signaling; therefore, the therapeutic response from tofacitinib in our patient is likely due, in part, to blockade of type 2 cytokine responses.^8^ Moreover, in recent studies, we found previously unrecognized JAK signaling in nerves that critically regulate AD-associated itch.^9^ Thus, the patient's improvement in terms of his pruritus may be caused by this newly discovered target of tofacitinib.

Our case highlights the potential value of using a novel and specific therapy to suppress multiple inflammatory dermatoses at once. A deeper understanding of the immunopathogenesis of each disorder allowed us to take a hypothesis-driven therapeutic approach to simultaneously treat 2 inflammatory skin disorders. Thus, with the advent of more targeted agents, as well as a better understanding of each patient's immune profile, clinical care will likely require a more personalized medicine approach in the near future.
